# Bridging the gap: tackling general and HPV vaccine hesitancy in rural and low-vaccination areas to improve HPV vaccine uptake

**DOI:** 10.3389/fpubh.2025.1702968

**Published:** 2026-01-27

**Authors:** Shillpa Naavaal, Joseph Boyle, Tegwyn Brickhouse, Askar Chukmaitov, Janaye Oliver, Bernard F. Fuemmeler

**Affiliations:** 1 Pediatric Dentistry and Dental Public Health and Policy, School of Dentistry, Virginia Commonwealth University, Richmond, VA, United States; 2 Department of Family Medicine and Population Health, School of Medicine, Virginia Commonwealth University, Richmond, VA, United States; 3 Department of Health Policy, School of Public Health, Virginia Commonwealth University, Richmond, VA, United States; 4 Independent Researcher, Richmond, VA, United States

**Keywords:** children and adolescents, human papillomavirus, low vaccination areas, rural areas, vaccine hesitancy

## Abstract

**Background:**

Despite strong efforts, HPV vaccine uptake remains low, especially in rural areas. This study examined general and HPV vaccine hesitancy among parents of children aged 9–17 in rural, low-vaccination areas, its link to HPV vaccination rates, and key factors influencing hesitancy.

**Methods:**

We surveyed parents from three counties in Virginia and collected information about their beliefs and opinions regarding adolescent vaccines, particularly the HPV vaccine, and their child's HPV vaccination status. General and HPV vaccine hesitancy were assessed using validated scales. Bayesian index logistic regression models were used to examine the relationship between each index and the child's HPV vaccination status, and also to identify the most important factor within each index.

**Results:**

The final analytical sample included 249 complete responses. The average reported child age was 12.4 years; 62.0% of parents reported that their child had received the HPV vaccine. In the adjusted general vaccine hesitancy model, hesitancy was significantly associated with a lower likelihood of a child's HPV vaccination (OR = 0.43, 95% CI: 0.26, 0.73), with perceived vaccine safety (weight = 0.363) the most influential factor estimated within the index. Similarly, in the adjusted HPV vaccine hesitancy model, hesitancy was significantly associated with a child's HPV vaccination (OR = 0.39, 95% CI: 0.20–0.72), with provider recommendation (weight = 0.198) the dominant estimated factor.

**Conclusion:**

Vaccine hesitancy remains a substantial challenge for HPV vaccination. Engaging providers to give strong vaccine recommendations and sharing information about vaccine safety, among other strategies, could help improve HPV vaccine uptake in rural and low-vaccination areas.

## Introduction

The Human Papillomavirus (HPV) is a prevalent infection. According to recent estimates, approximately 13 million individuals in the United States (US) are infected annually (1). The majority of HPV infections resolve on their own, but some high-risk strains can develop into cancers. HPV is linked to nearly 5% of all cancers, which include oropharyngeal, cervical, anal, penile, vulvar, and vaginal cancers, totaling over 37,000 cases in the US annually (2). HPV vaccines, which have been available since 2006 for girls and since 2011 for boys, are highly effective in preventing HPV infections and can prevent up to 90% of cancers caused by HPV (3). HPV vaccines have brought about a significant reduction in HPV infections and cervical pre-cancers, particularly decreases of 88% among teenage girls and 81% among young women (4). Additionally, the HPV vaccine provides long-lasting protection, with the best results achieved when it is administered before any exposure to the virus. Hence, it is recommended at 11–12 years of age, but can be given as early as 9 years of age. Over the last 2 decades, HPV vaccination rates have increased; however, they remain significantly below those of many other required adolescent vaccines and the Healthy People 2030 objective of achieving 80% up-to-date (UTD) vaccination coverage (5). In 2023, among adolescents aged 13–17, the HPV UTD percentage was 61.4% (64.0% in females and 59.0% in males), compared to 89.0% for Tdap (6).

Vaccine hesitancy—the delay or refusal to take a vaccine despite its availability—is a complex phenomenon and a significant public health challenge in the US. Despite the remarkable successes of immunization programs, compelling scientific evidence of high efficacy, robust safety, and proven public health impact, many parents are still hesitant to vaccinate their child against HPV (7). Data show that among parents of adolescents, reluctance to vaccinate stems from concerns about vaccine safety, side effects, longstanding mistrust in pharmaceutical companies and government institutions, misperceptions, and pervasive influence of misinformation (8, 9). To effectively monitor these behaviors, researchers have developed composite indices that quantitatively capture public sentiment toward vaccination. Validated instruments such as the Vaccine Hesitancy Scale—which distinguishes between a lack of confidence in vaccines and concerns regarding their risks—provide a robust basis for measuring these attitudes over time (10).

The majority of research on HPV vaccine hesitancy has historically been conducted in urban settings, where access to healthcare systems and larger sample sizes make studies more feasible. However, rural populations, despite showing lower vaccine uptake and higher burden of vaccine-preventable diseases, have been underrepresented. A systematic review examining area-level variation in HPV vaccination found that ZIP code tabulation area-level poverty, urbanicity/rurality, racial/ethnic composition, and health service region characteristics were strongly associated with HPV vaccination (11). Studies show that rural communities often experience unique barriers in accessing healthcare, including a shortage of providers, and reliable transportation, which are further complicated by a higher prevalence of low socioeconomic status in these communities (12).

Vaccine hesitancy, especially for HPV, can be particularly pronounced in rural areas, where several factors, including low health literacy and distinct cultural norms, combined with the factors mentioned above, can interact (12, 13). Data show that HPV vaccination is significantly lower in rural areas. For example, in Virginia, HPV UTD percentage is 50% vs. 62% in rural versus urban areas, respectively (14). The Student Immunization Status Report (SIS) concurs, revealing a low rate of HPV vaccination in rural Virginia counties (15). To improve HPV vaccination rates both nationally and locally, and to reduce cancer risk, it is crucial to increase vaccine uptake in regions with low coverage and high cancer incidence. Achieving this requires a deeper understanding of vaccine hesitancy in these communities, identifying the most influential factors and how both general and HPV vaccine hesitancy relate to HPV vaccine uptake.

Thus, the rationale of this study was to bridge this gap and examine the prevalence of general and HPV-specific vaccine hesitancy among parents and guardians of children aged 9–17 living in rural, low-vaccination areas. We examined the relationship between parental hesitancy and their child's HPV vaccination status and identified key factors influencing hesitancy.

## Methods

### Data source and study sample

This study was part of a larger mixed-method project investigating HPV vaccine hesitancy. It was carried out in three counties in Virginia, which were selected based on their low HPV vaccination rates and high incidence and mortality rates for cervical and oral cancers (16). For this study, parents and guardians were recruited through flyers and word of mouth and invited to participate in answering survey questions about their attitudes, beliefs, and opinions regarding adolescent vaccines, particularly the HPV vaccine, and their child's HPV vaccination status. The parents/guardians were eligible to participate in the study if they were above 18 years of age, had a child between 9 and 17 years of age who attended a school in one of three selected counties, or lived with them in one of those counties, and could complete the survey in English (hereon called as parents). Parents with multiple children were asked to complete the survey considering their youngest child. They could complete the survey online or using a paper questionnaire. Survey data were collected and managed using REDCap (Research Electronic Data Capture), a secure, web-based software platform designed to support data capture for research studies, hosted at Virginia Commonwealth University (17, 18). The data was collected between March 2021 and June 2021, and the study was approved by the Institutional Review Board at Virginia Commonwealth University.

### Survey description and covariates

Survey questions were selected and adapted from the previously validated questionnaires. The primary outcome variable was the child's HPV vaccination status, categorized as yes/no. The exposure variables included two vaccine hesitancy indexes. The general vaccine hesitancy was determined using the four-item short form of the Vaccine Confidence Scale (19). The HPV vaccine hesitancy was determined using the adapted nine-item Vaccine Hesitancy Scale (9). Response options for these questions consisted of the following: strongly agree, somewhat agree, somewhat disagree, and strongly disagree. The survey also collected sociodemographic information for the parent and child, including age, sex, race, ethnicity, and parent education, marital status, and household income. Participants who completed the survey were compensated and received a gift card.

### Statistical analysis

The sample's characteristics were summarized using descriptive statistics, employing means and standard deviations for continuous variables and counts and frequencies for categorical variables. The distributions of responses to all questions related to general and HPV vaccine hesitancy were examined individually and then used as an index in the model. We coded responses to the questions of (Strongly Disagree, Somewhat Disagree, Somewhat Agree, and Strongly Agree) into (3,2,1,0), in order that larger scores on the index would be in the direction of greater hesitation. Notably, we reverse-coded the last two questions in the HPV vaccine hesitancy index owing to their wording in the reverse direction.

Bayesian index logistic regression models were used to model the association between the correlated indices of hesitancy with the likelihood of the child's HPV vaccine receipt. These models are highly applicable to correlated socioeconomic and behavioral factors, and have demonstrated improved goodness of fit relative to other methods (20, 21). Their utility derives in part from a statistical prior distribution for the index weights that encodes some similarity for the weights but allows this to be updated by the degree of differing importance for index components shown in the data. They were used to estimate an overall effect for the index, as well as the relative importance weights for index components. These weights are standardized to be between 0 and 1 and sum to 1 for each index for interpretability. Starting with the unadjusted models, separate models were used to examine the general hesitancy index and the HPV hesitancy index, as well as both indices simultaneously. Then the adjusted models were fitted, adjusting for parent's age in groups (26–35 years, 36–45 years, 46+ years), race (Black or Other vs. reference White), education (less than college degree vs. reference college degree), marital status (not married vs. reference married), income groups, and child sex and age. Regression parameters were summarized using the mean and 95% credible interval (CI), which is the Bayesian equivalent of a confidence interval, and significance was determined at a 95% level of confidence. Model parameters were estimated using Markov chain Monte Carlo methods using the program Just Another Gibbs Sampler (JAGS), running two chains and burning in a large number of samples, then obtaining 10,000 iterations from the posterior distribution. Model convergence was evaluated using the Gelman-Rubin statistic, considering parameters to have converged if their value was less than 1.01. Model goodness of fit was compared using the deviance information criterion (DIC), a measure of model fit that penalizes model complexity. Lower values of the DIC indicate better fitting models, and differences of less than 5 in DIC indicate comparable fit (22). All parameters converged in all models. The analyses were performed using the R software, version 4.4.2.

## Results

The analytic study sample included responses from 249 parents. The survey received a total of 362 responses. The survey responses were excluded if there was an incomplete response on vaccine hesitancy questions (*n* = 103), a missing response on gender (*n* = 4), or county of interest (*n* = 2), and if the reported child's age was less than 5 years old (*n* = 4). Among the study sample, 154 parents reported their child had received HPV vaccination, and 95 had not [reporting “No” (*n* = 89), “Don't Know” (*n* = 5), or did not answer (*n* = 1)].

Table 1 summarizes sample characteristics. Most parents were between 36 and 45 years of age (76%) and male (51%). The majority of the study sample consisted of White (55%) and Black (41%) respondents. Forty-five percent of the parents reported having a college degree or higher, 47% reported some college education or an Associate's degree, and 80% were married. The majority of parents reported an annual family income of less than $75,000 (79%). The average reported age of a child was 12.4 years, and 61% of the children were male. Sixty-two percent of the parents reported that their child had received the HPV vaccine.

**Table 1:** Summary of sample characteristics.

**Variable**	**Frequency (%)**
**Age (in years)**	
26–35	27 (11)
36–45	188 (76)
46+	34 (17)
**Gender**	
Female	121 (49)
Male	128 (51)
**Race**	
White	137 (55)
Black/African American	103 (41)
American Indian	4 (2)
Multiracial	2 (1)
Asian	3 (1)
**Ethnicity**	
Hispanic/Latino	59 (24)
Not Hispanic/Latino	190 (76)
**Education**	
College degree, Post-graduate, or Professional	112 (45)
Some college, Associate's Degree and/or technical school	117 (47)
High School Graduate or GED	17 (7)
Less than High School	2 (1)
I choose not to answer	1 (0)
**Income**	
$75,000 or more	52 (21)
$50,000–$74,999	80 (32)
$25,000–$49,999	90 (36)
$15,000–$24,999	13 (5)
Less than $15,000	5 (2)
I choose not to answer	9 (4)
**Marital status**	
Married	198 (80)
Divorced/Separated	23 (9)
Widowed	13 (5)
Never Married	11 (4)
I choose not to answer	4 (2)
**Child gender**	
Male	153 (61)
Female	94 (38)
Did not answer	2 (1)
**Child age (mean age in years)**	12.4 (1.3)
**Child received HPV vaccine**	
Yes	154 (62)
No	95 (38)

Categorical variables are summarized with count (frequency). Continuous variables are summarized with mean (standard deviation).

Table 2 shows the distribution of responses to the vaccine hesitancy questions. Parents generally responded favorably with respect to general vaccine questions. More than 70% of parents responded with some degree of agreeability that vaccines were necessary to protect the health of adolescents (82%), that vaccines could prevent diseases they were intended to (79%), that vaccines were safe (80%), and that lack of vaccination could lead to spread of disease (71%). However, there was a greater variation in responses favoring the HPV vaccine (41% to 84%). The majority of parents (84%) agreed that the information about the HPV vaccine received from healthcare providers was reliable and trustworthy. However, only 41% of parents reported that the HPV vaccine had existed long enough to ensure safety, and 47% were not concerned about the side effects of the HPV vaccine.

**Table 2:** Distribution of responses to vaccine hesitancy questions.

**Index**	**Question**	**Strongly disagree**	**Somewhat disagree**	**Somewhat agree**	**Strongly agree**
General	Vaccines are *necessary* to protect the health of adolescents.	24 (10)	22 (9)	106 (43)	97 (39)
	Vaccines are good in *preventing* the diseases they are intended to prevent.	21 (8)	31 (12)	67 (27)	130 (52)
	Vaccines are *safe*.	10 (4)	42 (17)	91 (37)	106 (43)
	Adolescents who are not vaccinated can get a disease and can *spread* the disease to others.	13 (5)	60 (24)	72 (29)	104 (42)
HPV	The information I receive about the HPV vaccine from my child's health care provider is *reliable* and trustworthy.	12 (5)	28 (11)	121 (49)	88 (35)
	The HPV vaccine is *effective*.	37 (15)	31 (12)	75 (30)	106 (43)
	Getting the HPV vaccine is a good way to protect my child from developing *HPV-related cancers*.	12 (5)	59 (24)	68 (27)	110 (44)
	The HPV vaccine is *beneficial* for my child.	9 (4)	54 (22)	87 (35)	99 (40)
	I do/did what my child's health provider *recommends* about the HPV vaccine.	8 (3)	45 (18)	98 (39)	98 (39)
	The HPV vaccine is important for my child's *health*.	10 (4)	43 (17)	88 (35)	108 (43)
	Having my child get the HPV vaccine is important for the health of others in my *community*.	28 (11)	40 (16)	88 (35)	93 (37)
	The HPV vaccine has not been around long enough to be sure it is *safe*.	37 (15)	64 (26)	81 (33)	67 (27)
	I am concerned about the *serious side effects* of the HPV vaccine.	46 (18)	73 (29)	87 (35)	43 (17)

Numbers in the table denote count (percentage).

The unadjusted associations between the vaccine indices and the likelihood of a child receiving HPV vaccination are summarized in Table 3, presented separately for each hesitancy measure. The general vaccine hesitancy index was significantly associated with a child's HPV vaccination status, where greater parental hesitation was associated with a lower likelihood of a child's HPV vaccination (OR = 0.61, 95% CI: 0.41, 0.90). Within this index, the most important factor was the perception of vaccines' safety, receiving the largest estimated importance weight of 0.407. The HPV vaccination hesitancy index was also significantly associated with a child's vaccine status (OR = 0.58, 95% CI: 0.35, 0.97). Within this index, the most important factor was the perception of following the child's healthcare provider's recommendations regarding the HPV vaccine, receiving the largest estimated importance weight of 0.191. The general and HPV-specific indices provided very similar model fit. There was a strong positive correlation between the two indices (*r* = 0.81); therefore, both indices were not tested together in the unadjusted models.

**Table 3:** Summary of unadjusted models examining the relationship between vaccine hesitancy indices and children's HPV vaccination status and estimated importance weights for each included indicator within the index.

**Index**	**Index type**	**Index odds ratio (95% credible interval)**	
		General	HPV
	General	0.61 (0.41, 0.90)	–
HPV	–	0.58 (0.35, 0.97)
**Variable**	**Index type**	**Estimated importance weights**	
Vaccines are *necessary* to protect the health of adolescent	General	0.085	–
Vaccines are good in *preventing* the diseases they are intended to prevent.		0.229	–
Vaccines are *safe*.		0.407	–
Adolescents who are not vaccinated can get a disease and can *spread* the disease to others.		0.279	–
The information I receive about the HPV vaccine from my child's health care provider is *reliable* and trust.	HPV	–	0.123
The HPV vaccine is *effective*.		–	0.065
Getting the HPV vaccine is a good way to protect my child from developing *HPV-related cancers*.		–	0.085
The HPV vaccine is *beneficial* for my child.		–	0.161
I do/did what my child's health provider *recommends* about the HPV vaccine.		–	0.191
The HPV vaccine is important for my child's *health*.		–	0.174
Having my child get the HPV vaccine is important for the health of others in my *community*.		–	0.070
The HPV vaccine has not been around long enough to be sure it is *safe*.		–	0.050
I am concerned about the *serious side effects* of the HPV vaccine.		–	0.081
Deviance Information Criterion (DIC)		328.3	329.4

Importance weights sum to 1 for each index. Lower values of DIC indicate better-fitting models.

Table 4 presents the adjusted associations (conditional on model covariates) between the vaccine indices and the likelihood of a child receiving an HPV vaccination, controlling for parent- and child-level covariates. The associations were in the same direction and found to be slightly stronger in magnitude when adjusting for these factors. In the general vaccination hesitancy index model, hesitancy was significantly associated with the child's HPV vaccination status (OR = 0.43, 95% CI: 0.26, 0.73). Within this index, the most important factor was the safety of the vaccines, receiving the largest estimated importance weight of 0.363, followed by the factor that not vaccinating can spread disease to others (0.358). In the HPV vaccination hesitancy index model, hesitancy was significantly associated with the child's vaccine status (OR = 0.39, 95% CI: 0.20, 0.72). Within this index, the most important factor was the perception of doing what the child's health provider recommends regarding the HPV vaccine, receiving the largest estimated importance weight of 0.198, followed closely by the factor that the vaccine is beneficial, with a weight of 0.187. In both models, holding other factors constant, Black parents (relative to white parents) and parents in a household reporting a household income of $15,000–$24,999 (relative to a household income of above $75,000) were significantly less likely to report child's HPV vaccination status (summary of covariates given in Supplemental material). The general and HPV-specific indices provided very similar model fit.

**Table 4:** Summary of adjusted models examining the relationship between vaccine hesitancy indices and children's HPV vaccination status and estimated importance weights for each included indicator within the index.

**Index**	**Index Type**	**Index odds ratio (95% credible interval)**	
		General	HPV
	General	0.43 (0.26, 0.73)	–
HPV	–	0.39 (0.20, 0.72)
**Variable**	**Index type**	**Estimated importance weights**	
Vaccines are *necessary* to protect the health of adolescents.	General	0.074	–
Vaccines are good in *preventing* the diseases they are intended to prevent.		0.205	–
Vaccines are *safe*.		0.363	–
Adolescents who are not vaccinated can get a disease and can *spread* the disease to others.		0.358	–
The information I receive about the HPV vaccine from my child's healthcare provider is *reliable* and trustworthy.	HPV	–	0.161
The HPV vaccine is *effective*.		–	0.055
Getting the HPV vaccine is a good way to protect my child from developing *HPV-related cancers*.		–	0.087
The HPV vaccine is *beneficial* for my child.		–	0.187
I do/did what my child's health provider *recommends* about the HPV vaccine.		–	0.198
The HPV vaccine is important for my child's *health*.		–	0.159
Having my child get the HPV vaccine is important for the health of others in my *community*.		–	0.059
The HPV vaccine has not been around long enough to be sure it is *safe*.		–	0.036
I am concerned about the *serious side effects* of the HPV vaccine.		–	0.059
Deviance Information Criterion (DIC)		321.6	322.3

Importance weights sum to 1 for each index. Lower values of DIC indicate better-fitting models. The model is controlled for sociodemographic covariates.

## Discussion

The study findings highlight the persistent challenges to HPV vaccination, especially in rural areas, and build upon prior work by utilizing Bayesian index logistic regression models, offering a novel and robust approach to quantifying vaccine hesitancy and its multifaceted dimensions. The study demonstrates that both general vaccine hesitancy and HPV-specific hesitancy are significantly associated with lower HPV vaccine uptake among our sample of adolescents from areas with low vaccination uptake. Notably, vaccine safety (in general) and trust in healthcare provider recommendations (specific to HPV) emerged as the most influential factors shaping parental decision-making, as indicated by respective hesitancy indices, though certain other specific factors also contributed to these indices. These findings are consistent with previous research indicating that vaccine confidence plays a pivotal role in immunization behaviors (8, 10). Our findings also suggest a particularly strong influence of healthcare provider recommendations in rural communities. Previous studies have found that provider shortage and a lack of stronger recommendations are significant factors driving lower vaccination rates (23).

Prior research has shown that rural populations face additional barriers, including limited healthcare access, provider shortages, socioeconomic constraints, and cultural beliefs, which contribute to vaccine hesitancy (11, 24). Our findings align with these patterns and further emphasize the role of structural determinants, including income disparities and racial differences, in shaping vaccine uptake. Specifically, we found that Black parents and lower-income households were significantly less likely to report HPV vaccination for their children. Given that 41% of the study sample identified as Black and 43% of families reported annual incomes below $50,000, addressing hesitancy among these populations is crucial to improving vaccine uptake.

Addressing vaccine hesitancy requires both well-designed system-level and pragmatic, community-centered solutions. School-entry vaccination requirements have proven to be one of the most effective public health tools for vaccine-preventable diseases (25). However, a study found that policies with ambiguous language, loose mandates, or broad exemption options contribute to public confusion and lower vaccine uptake, especially in rural areas (26). Evidence-based interventions such as provider-driven education, pharmacist-led outreach initiatives, and targeted public health campaigns have been shown to improve HPV vaccine acceptance (27). Expanding access through mobile clinics and school-based vaccination programs may also help mitigate logistical barriers. Additionally, dental providers play a crucial role in HPV vaccine advocacy, as they frequently encounter adolescent patients and their parents during routine oral health visits and are trusted messengers (28). Given the established link between HPV and oropharyngeal cancers, dental professionals are uniquely positioned to educate patients about HPV risks and the benefits of vaccination. Integrating HPV vaccine discussions into dental visits, training dental providers to address vaccine hesitancy, and incorporating vaccination referrals within dental clinics could significantly contribute to improved uptake (29, 30). Strengthening provider-patient communication by fostering trust and transparency across medical and dental care settings can also play a crucial role in addressing parental concerns about vaccine safety and efficacy and in addressing the provider shortage barrier.

This study offers several important lessons for future efforts aimed at increasing HPV vaccination rates in areas with low vaccination rates. First, interventions should prioritize addressing parental vaccine safety concerns, as these perceptions were the strongest predictors of hesitancy. Public health campaigns using effective communication strategies should emphasize the extensive research supporting HPV vaccine safety and its role in preventing HPV-related cancers. Second, engaging and leveraging trusted local healthcare professionals and community leaders (such as school administrators, religious leaders, and parents) to disseminate accurate vaccine information and effective recommendations may be instrumental in countering misinformation and reinforcing confidence in vaccination. Strong, consistent, and confident recommendations using an announcement approach by health providers have been found to increase HPV vaccine uptake among adolescents (31). Third, incorporating culturally tailored and regionally specific messaging tailored to rural populations can enhance engagement and trust. Messaging strategies could include testimonials from local community members who have successfully vaccinated their children, reinforcing the vaccine's importance through relatable experiences. Additionally, healthcare providers, including dental professionals and pharmacists, should receive training on culturally competent communication techniques to address concerns in a manner that aligns with community values and beliefs (32). Community-based partnerships, including collaborations with local schools, faith-based organizations, and medical and dental provider organizations, could enhance outreach and education efforts to parents and children (33). Additionally, policy-level efforts such as clearer and more enforceable school vaccination mandates, coupled with value-based incentives for dental and medical healthcare providers to promote HPV vaccination, could contribute to higher uptake rates.

Despite its contributions, this study has some limitations. The sample was drawn from three specific rural counties in Virginia, which may limit the generalizability of our findings to other regions and states. The self-reported survey responses may be subject to social desirability and recall bias. Additionally, this study was conducted in 2021 when the COVID-19 pandemic was highly salient across the country, and vaccination discussions and debates were prominent. Views related to vaccines may have shifted since then and are important to continually assess and analyze. Also, it could be possible that overall summary measures such as odds ratios could differ if the relationship between vaccine index scale components and likelihood of vaccination differs strongly between different groupings of adjacent responses (e.g., from Somewhat Disagree to Somewhat Agree and Somewhat Agree to Strongly Agree). A larger number of possible responses could be beneficial to this end, though the scales we utilized were validated instruments. However, the study's strengths include its use of validated hesitancy scales and advanced statistical modeling (incorporating Bayesian updating for the index effect and weight components), as well as adjustments for important individual-level factors, which enhance the reliability of our results. The dynamic nature of vaccine hesitancy requires continuous monitoring and adaptation of strategies. Future research should explore longitudinal changes in vaccine hesitancy, as well as the effectiveness of targeted interventions designed to address the specific barriers identified in this study. In our study sample, the general and HPV-specific indices were highly correlated, providing a very similar model fit. This may suggest a similar latent construct underlying both indices. Future research can also explore factor analysis methods to uncover such constructs.

## Conclusions

HPV vaccine hesitancy remains a significant barrier to achieving optimal vaccination coverage in rural communities. Achieving high HPV vaccination coverage, particularly in vulnerable rural populations, is not merely a matter of vaccine availability but a complex interplay requiring sustained commitment to addressing ongoing barriers, fostering trust, engaging trusted messengers and implementing tailored, evidence-based interventions. By addressing key concerns surrounding vaccine safety, enhancing provider communication, and expanding accessible vaccination services, public health initiatives can make meaningful strides in HPV vaccine uptake, address the root causes of hesitancy, and improve health outcomes in underserved populations.

## Data Availability

The datasets presented in this article are not readily available because according to the IRB protocol, data will not be shared with anyone outside the research team. If requested, the amendment will have to be submitted to IRB to share the data. Requests to access the datasets should be directed to naavaals@vcu.edu.
