# Supplementary material for: Bridging the gap: tackling general and HPV vaccine hesitancy in rural and low-vaccination areas to improve HPV vaccine uptake
Source: Front Public Health. 2026 Jan 27;13:1702968. doi: 10.3389/fpubh.2025.1702968 (PMC12888034; doi:10.3389/fpubh.2025.1702968)
Supplement: Supplementary file 1 [file Table_1.docx]

Supplementary Table 1. Adjusted model estimates for covariates

| Index: General Hesitancy |  |  |  |
| --- | --- | --- | --- |
| **Variable** | Odds Ratio | Lower CI | Upper CI |
| Parent: Age > 35 and Age <= 45 | 2.56 | 0.97 | 6.83 |
| Parent: Age > 45 | 1.56 | 0.48 | 5.51 |
| Parent: Male | 1.18 | 0.55 | 2.55 |
| Parent race: Black | **0.47** | **0.25** | **0.90** |
| Parent race: Other | 1.81 | 0.26 | 12.92 |
| Education: less than college | 0.90 | 0.46 | 1.76 |
| Marital status: not married | 0.65 | 0.27 | 1.55 |
| Household income: $50k-$75k | 0.44 | 0.19 | 1.03 |
| Household income: $25k-$50k | 0.43 | 0.18 | 1.07 |
| Household income: $15k-$25k | **0.06** | **0.01** | **0.33** |
| Household income: <$15k/did not answer | 0.31 | 0.05 | 1.87 |
| Child gender: Female | 0.97 | 0.46 | 1.97 |
| Child age | 1.25 | 0.93 | 1.62 |

| Index: HPV Hesitancy |  |  |  |
| --- | --- | --- | --- |
| **Variable** | Odds Ratio | Lower CI | Upper CI |
| Parent: Age > 35 and Age <= 45 | 2.09 | 0.78 | 5.82 |
| Parent: Age > 45 | 1.37 | 0.41 | 4.92 |
| Parent: Male | 1.24 | 0.57 | 2.75 |
| Parent race: Black | **0.47** | **0.25** | **0.89** |
| Parent race: Other | 2.09 | 0.33 | 15.49 |
| Education: less than college | 0.87 | 0.43 | 1.65 |
| Marital status: not married | 0.66 | 0.28 | 1.62 |
| Household income: $50k-$75k | 0.48 | 0.20 | 1.11 |
| Household income: $25k-$50k | 0.44 | 0.18 | 1.11 |
| Household income: $15k-$25k | **0.07** | **0.01** | **0.35** |
| Household income: <$15k/did not answer | 0.31 | 0.05 | 2.06 |
| Child gender: Female | 0.92 | 0.46 | 1.93 |
| Child age | 1.24 | 0.96 | 1.62 |
